# Highly sensitive and rapid determination of* Mycobacterium leprae* based on real-time multiple cross displacement amplification

**DOI:** 10.1186/s12866-023-03004-7

**Published:** 2023-09-28

**Authors:** Junfei Huang, Yi Tong, Yijiang Chen, Xinggui Yang, Xiaoyu Wei, Xu Chen, Jinlan Li, Shijun Li

**Affiliations:** 1 Laboratory of Infectious Disease of Experimental Center, Guizhou Provincial Center for Disease Control and Prevention, Guiyang, Guizhou 550004 People’s Republic of China; 2 The Second Affiliated Hospital, Guizhou University of Traditional Chinese Medicine, Guiyang, Guizhou 550003 People’s Republic of China; 3 Tuberculosis Control Institute, Guizhou Provincial Center for Disease Control and Prevention, Guiyang, Guizhou 550004 People’s Republic of China; 4 School of Public Health, Guizhou Medical University, Guiyang, Guizhou 550025 People’s Republic of China

**Keywords:** *Mycobacterium leprae*, Detection, Real-time, Multiple cross displacement amplification, Endonuclease

## Abstract

**Background:**

*Mycobacterium leprae* (ML) is the pathogen that causes leprosy, which has a long history and still exists today. ML is an intracellular mycobacterium that dominantly induces leprosy by causing permanent damage to the skin, nerves, limbs and eyes as well as deformities and disabilities. Moreover, ML grows slowly and is nonculturable in vitro. Given the prevalence of leprosy, a highly sensitive and rapid method for the early diagnosis of leprosy is urgently needed.

**Results:**

In this study, we devised a novel tool for the diagnosis of leprosy by combining restriction endonuclease, real-time fluorescence analysis and multiple cross displacement amplification (E-RT-MCDA). To establish the system, primers for the target gene RLEP were designed, and the optimal conditions for E-RT-MCDA at 67 °C for 36 min were determined. Genomic DNA from ML, various pathogens and clinical samples was used to evaluate and optimize the E-RT-MCDA assay. The limit of detection (LoD) was 48.6 fg per vessel for pure ML genomic DNA, and the specificity of detection was as high as 100%. In addition, the detection process could be completed in 36 min by using a real-time monitor.

**Conclusion:**

The E-RT-MCDA method devised in the current study is a reliable, sensitive and rapid technique for leprosy diagnosis and could be used as a potential tool in clinical settings.

**Supplementary Information:**

The online version contains supplementary material available at 10.1186/s12866-023-03004-7.

## Background

Leprosy is a chronic granulomatous disease triggered by infection with *Mycobacterium leprae* (ML). ML is an intracellular mycobacterium that permanently damages the skin, nerves, limbs eyes and even causes deformities and disabilities [1–3]. Although the World Health Organization (WHO) had set a target to interrupt the transmission of leprosy worldwide by 2020, 127,558 new cases emerged in that year, most of which were observed in developing countries [1, 4]. To date, multidrug therapy (MDT) has been the main approach to controlling the disease [4, 5]. However, due to a range of personal, psychosocial, economic, medical, and health service factors, a significant number of patients become irregular and drop out of MDT [4], and leprosy remains a public health problem in many areas of the world. Thus, a reliable and rapid detection tool for the early diagnosis of leprosy is urgently needed.

ML is a slow-growing organism and cannot be cultured in vitro, although it can proliferate well in the living body. In general, the bacterium is usually cultured in the foot pads of mice and armadillos to obtain a massive amount of ML [2, 6]. Currently, the approaches for ML detection include histopathological examination, acid-fast bacilli (AFB) microscopy, serological testing and molecular biological detection [7–9]. However, diagnosis of early stage leprosy based on clinical symptoms and slit skin smear (SSS) is insensitive [8]. In addition, similar to other diseases caused by mycobacterial infection, leprosy is poorly diagnosed by clinical criteria and AFB microscopy [8, 10]. Moreover, serological tests including recombinant proteins and semisynthetic glycoproteins (e.g., phenolic glycolipid I, LID-1, LID-ND-O, and antigen 85B) have been widely used to diagnose leprosy [11]. However, these methods lack sensitivity and specificity [7]. Instead, molecular biological methods show great advantages with high sensitivity and specificity in the rapid identification and diagnosis of early cases [5, 7, 8].

To date, molecular techniques such as conventional PCR [8, 9], real-time quantitative PCR (RT‒PCR) [12], and loop-mediated isothermal amplification (LAMP) [13, 14], have been used for the rapid detection of ML. PCR-based assays are konwn to rely on the cycle procedure of denaturation, annealing and extension [8, 9, 12]. However, this amplification procedure usually takes 2 h to complete, which is time-consuming. Furthermore, the conventional PCR method requires additional time to perform agarose gel electrophoresis and UV illumination examination [8, 9]. Fortunately, isothermal amplification approach has been developed to overcome the shortages of PCR-based techniques. These approaches include loop-mediated isothermal amplification (LAMP) [15], recombinase polymerase amplification (RPA) [16], and multiple cross displacement amplification (MCDA) [17], which have shown outstanding capabilities in the diagnosis of various pathogens (e.g., *Salmonella, Listeria*, *M. tuberculosis* and *SARS-CoV-2*) [18–21]. Currently, the products of isothermal amplification are validated through conventional agarose gel electrophoresis, real-time fluorescence readout, visual indicator reagents (e.g., malachite green [MG), real-time turbidity and nanoparticle-based lateral flow biosensors (LFBs) [13, 19, 21, 22].

Here, by combining Nb.*BsrDI* (a restriction endonuclease), MCDA and real-time fluorescence analysis, we developed a novel method, termed E-RT-MCDA, for the diagnosis of leprosy. In the established system, the target gene (RLEP) was amplified in an MCDA reaction tube, Nb.*BsrDI* was uesd to recognise and digest the base site, and real-time PCR was used to collect the fluorescence signal. Additionally, the purified genomic DNA of ML was employed to investigate the method’s efficiency and sensitivity, and different pathogens and clinical samples were used to evaluate the specificity and feasibility of the E-RT-MCDA method.

## Results

### Confirmation and detection of E-RT-MCDA products

**Fig. 1:**
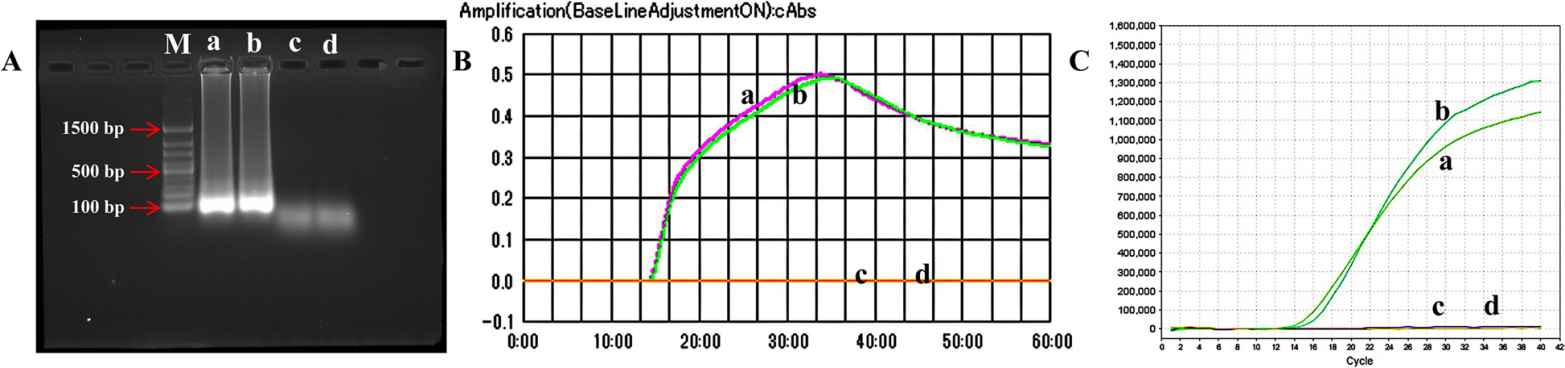
Confirmation and detection of MCDA products. **A** Agarose gel electrophoresis for MCDA products. **B** Real-time turbidity detection for the MCDA reaction. **C** The real-time fluorescence assay for MCDA products. **a** ML genomic DNA at 1.52 × 10^5^ fg per mixture. **b** ML genomic DNA at 3.04 × 10^4^ fg per mixture. **c** The negative control (NC) containing the *M. tuberculosis* genomic DNA. **d** Double-distilled water (DW) used as the blank control (BC)

To confirm the E-RT-MCDA reaction, we employed agarose gel electrophoresis, a real-time turbidimeter (LA-500) and real-time fluorescence to detect the products. The ML genomic DNA (including 1.52 × 10^5^ fg per mixture and 3.04 × 10^4^ fg per mixture) was used in these assays. *M. tuberculosis* genomic DNA was used as the negative control (NC), and double-distilled water (DW) was used as the blank control (BC). The results revealed that in the agarose gel electrophoresis experiment, the positive mixtures which containing DNA templates of ML showed the enriched ladders (approximately 100–200 bp) under the UV light, and the NC and BC were invisible (Fig. 1A). Moreover, in the real-time turbidity assay, the E-RT-MCDA amplification products accumulated the turbidity value, while the turbidity of the NC and BC were not increased (Fig. 1B). For the real-time fluorescence detection, the reaction products were tested by real-time PCR. The amplification product curves were positive, whereas the NC and BC curves were not (Fig. 1C). Thus, using the three approaches above, indicated that E-RT-MCDA worked well by detecting the RLEP gene.

### Optimization of application temperature and time for the E-RT-MCDA assay

**Fig. 2:**
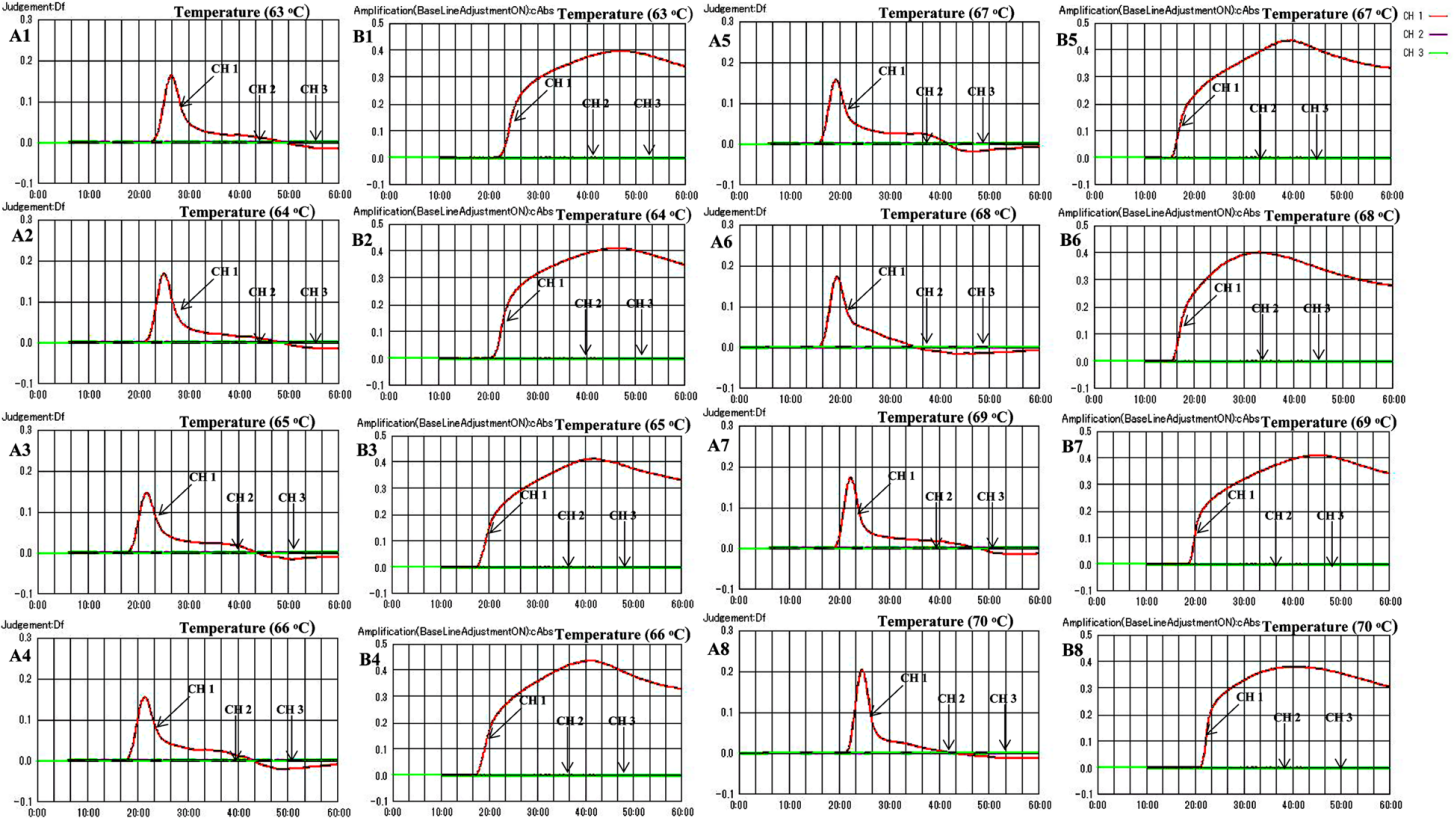
Optimal reaction temperature for the ML MCDA assay. ML genomic DNA was subjected to the standard MCDA reaction at the level of 1.52 × 10^5^ fg per mixture, and monitored by real-time turbidimetry. Different reaction temperatures were then used for the tests, ranging from 63 to 70 °C, with intervals of 1 °C. The threshold value of turbidity ≥ 0.1 was considered positive amplification. (A1-A8) Real-time turbidity kinetic graphs. (B1-B8) The accumulation turbidity kinetic graphs. (CH1) The ML genomic DNA at 1.52 × 10^5^ fg per mixture. (CH2) Negative control (NC) containing *M. tuberculosis* genomic DNA. (CH3) Double-distilled water (DW) used as the blank control (BC)

To further optimize the reaction conditions, we adjusted the temperature parameter by setting up a temperature gradient ranging from 63 to 70 °C (with intervals of 1 °C). The ML genomic DNA was subjected to the E-RT-MCDA reaction at the level of 1.52 × 10^5^ fg per mixture, and the amplicons were detected although the real-time turbidimeter. The data showed that the reaction was fast with highest turbidity value at 67 °C (Fig. 2). In addition, the turbidity value results also indicated that the saturation time point was at 36 min. Therefore, we determined that the application temperature and time of E-RT-MCDA was 67 °C for 36 min.

### Sensitivity of the E-RT-MCDA assay

**Fig. 3:**
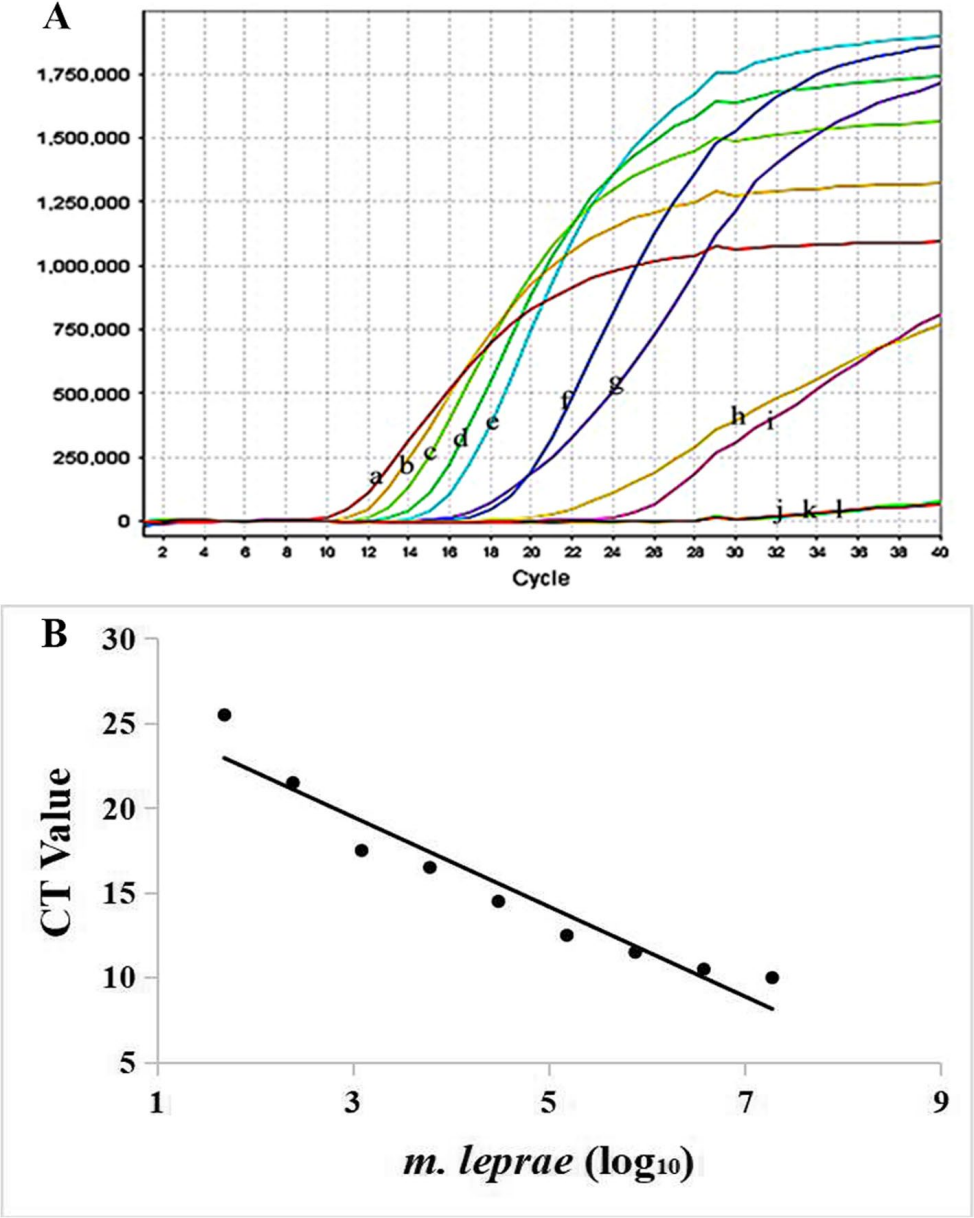
Analytical sensitivity of the E-RT-MCDA assay targeting the RLEP gene. **A** The serial fivefold dilutions (a 1.9 × 10^7^ fg/µl, b 3.8 × 10^6^ fg/µl, c 7.6 × 10^5^ fg/µl, d 1.52 × 10^5^ fg/µl, e 3.04 × 10^4^ fg/µl, f 6.08 × 10^3^ fg/µl, g 1.22 × 10^3^ fg/µl, h 2.43 × 10^2^ fg/µl, i 4.86 × 10^1^ fg/µl, j 9.73 fg/µl, and k 1.95 fg/ul) of ML DNA shall be tested according to the standard E-RT-MCDA. (l) Double-distilled water (DW) was used as a blank control (BC). **B** The CT was plotted against the DNA concentration of the positive reactions. The ordinate is the number of PCR cycles at the threshold and the abscissa is the number of ML DNA(log10)

Using the screened temperature and time conditions, we next investigated the sensitivity of E-RT-MCDA by serial genomic DNA dilutions (1.9 × 10^7^ fg-1.95 fg per microlitre) of ML genomic DNA. The results showed that the genomic DNA concentrations ranging from 1.9 × 10^7^ fg to 4.86 × 10^1^ fg per reaction were positive, while concentrations below 48.64 fg per reaction and the BC were not detected. Therefore, the LoD of the RLEP gene in this assay was 48.64 fg of genomic DNA per reaction (Fig. 3A). Moreover, the CT value was plotted against the DNA concentration of the reactions according to the detection range from 1.9 × 10^7^ fg to 4.86 × 10^1^ fg (Fig. 3B).

### Analytical specificity of the E-RT-MCDA assay

**Fig. 4:**
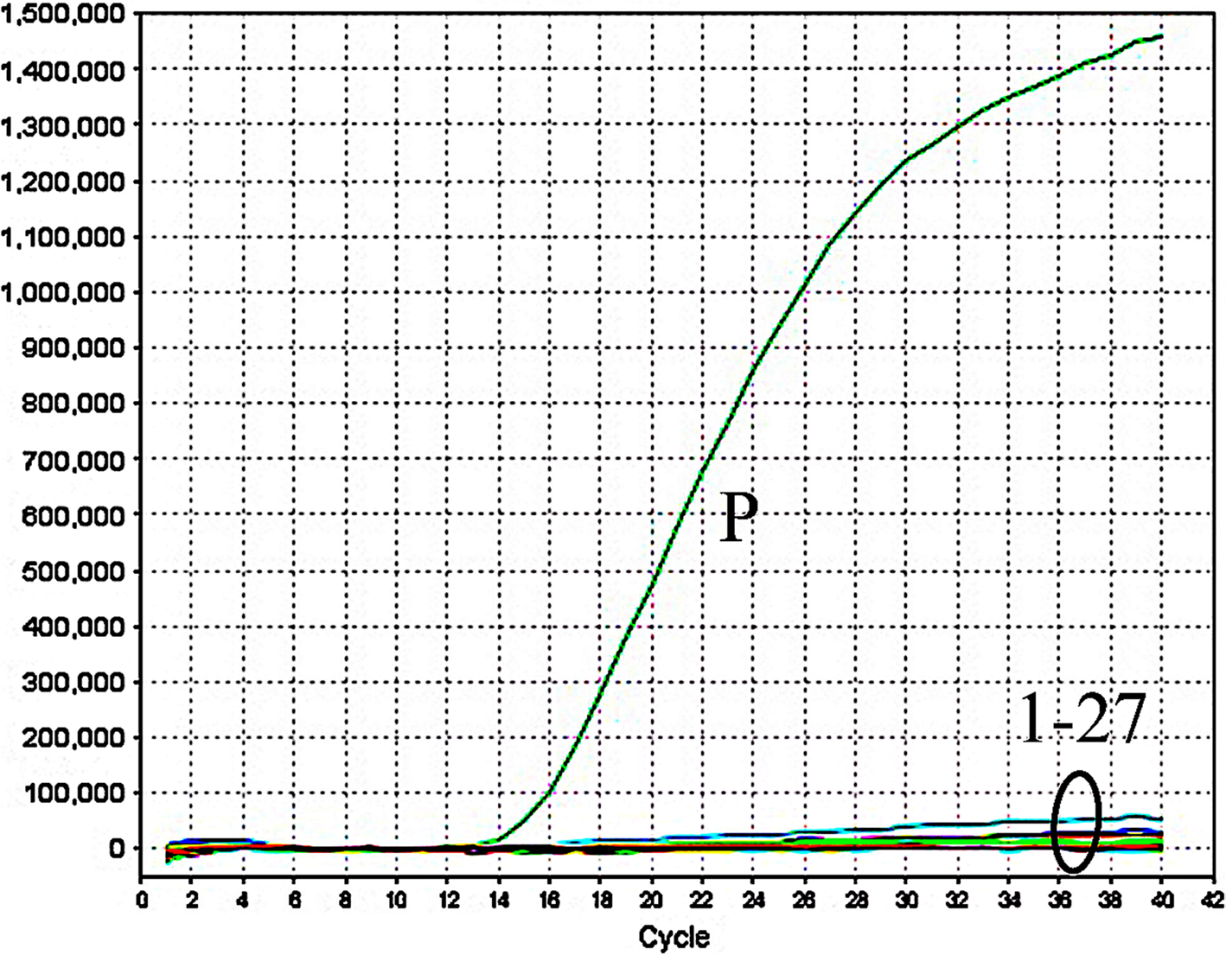
Specificity of the E-RT-MCDA assay for different pathogens. The E-RT-MCDA assays are performed using different DNA templates and monitored using a real-time fluorescence detector. Curve P, strain of ML. Curves 1–27, strains of *M. tuberculosis*, *M. bovis, M. neoaurum, M. intracellulare, M. chelonae, M. vaccae, M. marinum, M. gilvum, M. microti, M. ulcerans, M. phlei, M. scrofulaceum, M. kansassi, M. triviale, M. gastri, M. malmoense, Klebsiella pneumoniae, Bacillus anthracis, Pseudomonas aeruginosa, Brucella melitensis, Streptococcus pneumoniae, Salmonella, Haemophilus influenzae,Staphylococcus aureus, Shigella, Streptococcus suis*, and double-distilled water

It is also important to understand the specificity of the novel E-RT-MCDA method. Specificity was assessed by analyzing the genomic DNA of 26 pathogenic bacterial strains (Table 1). Furthermore, the ML genomic DNA was used as a positive control (PC), and DW was used as a blank control (BC). As expected, only the PC group showed an amplification curve, while the DNA of 26 other bacterial strains and the BC group were negative, indicating that the specificity of the E-RT-MCDA methodology was as high as 100% (Fig. 4).

**Table 1:** The details of the pathogenic bacteria

Bacteria	Source of strains	No. of strains	E-RT-MCDA results
*M. leprae*	Isolate strains (CAMS)	1	P
*M. tuberculosis*	H37Rv, ATCC27294	1	N
*M. bovis*	ATCC 19210	1	N
*M. neoaurum*	ATCC25795	1	N
*M. intracellulare*	ATCC13950	1	N
*M. chelonae*	ATCC14472	1	N
*M. vaccae*	ATCC15483	1	N
*M. marinum*	ATACC927	1	N
*M. gilvum*	ATCC43909	1	N
*M. microti*	ATCC19422	1	N
*M. ulcerans*	ATCC19423	1	N
*M. phlei*	ATCC11758	1	N
*M. scrofulaceum*	ATCC19981	1	N
*M. kansassi*	ATCC12478	1	N
*M. triviale*	ATCC23292	1	N
*M. gastri*	ATCC15754	1	N
*M. malmoense*	ATCC29571	1	N
*Klebsiella pneumoniae*	Isolate strains (GZCDC)	1	N
*Bacillus anthracis*	Isolate strains (GZCDC)	1	N
*Pseudomonas aeruginosa*	Isolate strains (GZCDC)	1	N
*Brucella melitensis*	Isolate strains (GZCDC)	1	N
*Streptococcus pneumoniae*	Isolate strains (GZCDC)	1	N
*Nontyphoidal Salmonella*	Isolate strains (GZCDC)	1	N
*Haemophilus influenzae*	Isolate strains (GZCDC)	1	N
*Staphylococcus aureus*	Isolate strains (GZCDC)	1	N
*Shigella dysenteriae*	Isolate strains (GZCDC)	1	N
*Streptococcus suis*	Isolate strains (GZCDC)	1	N

*Abbreviations*: *CAMS* Chinese Academy of Medical Sciences, *ATCC* American Type Culture Collection, *GZCDC* Guizhou Provincial Center for Disease Control and Prevention, *P* Positive, *N* Negative

### Practical application of the E-RT-MCDA assay for clinical samples

To explore the clinical application of the E-RT-MCDA system in leprosy, both conventional PCR and E-RT-MCDA methods were employed to test the samples, individually. A total of 45 leprosy samples including skin tissue fluid samples and skin tissue samples, were classified into 7 cases of paucibacillary (PB) and 38 cases of multibacillary (MB) according to the clinical characteristics (Table S1). For the different sample types, the conventional PCR showed 9 (60.00%) positive results in skin tissue fluid (out of 15 samples) and 27 (90.00%) positive results in skin tissue (out of 30 samples), whereas E-RT-MCDA showed 13 (86.67%) positive results in skin tissue fluid and 29 (96.67%) positive results in skin tissue. On the other hand, the conventional PCR results indicated that 4 (57.14%) PB (out of 7 cases) and 32 (84.21%) MB (out of 38 cases) samples were positive, while E-RT-MCDA indicated 5 (71.43%) PB-positive and 37 (97.37%) MB-positive samples. In summary, the conventional PCR shad an overall positive rate of 80.00% (36/45) (Fig. S1), and E-RT-MCDA had an overall positive rate of 93.33% (42/45) (Table 2). The E-RT-MCDA assay had an excellent detection capabilities in clinical samples of leprosy compared to the conventional method.

**Table 2:** Detection of clinical leprosy samples

Leprosy samples	Methods			
Types/Classification	PCR		E-RT-MCDA	
	Positive	Negative	Positive	Negative
Skin tissue fluid (N_1_ = 15)	9 (60.00%)	6 (40.00%)	13 (86.67%)	2 (13.33%)
Skin tissue (N_2_ = 30)	27 (90.00%)	3 (10.0%)	29 (96.67%)	1 (3.33%)
PB (N_3_ = 7)	4 (57.14%)	3 (42.86%)	5 (71.43%)	2 (28.57%)
MB (N_4_ = 38)	32(84.21%)	6 (15.79%)	37 (97.37%)	1 (2.63%)
Total (N = 45)	36 (80.00%)	9 (20.00%)	42 (93.33%)	3 (6.67%)

*Abbreviations*: *PCR* Polymerase chain reaction, *E-RT-MCDA* Multiple cross displacement amplification combined with endonuclease restriction-mediated real-time PCR, *PB*, Paucibacillary, *MB* Multibacillary, *N* Number

## Discussion

Leprosy continues to be a public health concern worldwide, with a high incidence in areas of poverty [1, 4]. Due to the slow-growing and nonculturable in vitro characteristics of the pathogen [2, 6], the development of an accurate, sensitive and rapid tool for the detection of ML is urgently needed*.* Many reports have shown that PCR-based techniques (including conventional PCR, multiplex PCR and RT‒PCR) have an excellent ability to diagnose leprosy [5]. Although they are successful in detecting MB cases, they tend to have a low detection rate in PB cases [5]. Moreover, PCR-based detection depends on the cycle procedures of denaturation, annealing, and extension, which is time-consuming. Therefore, a highly sensitive and rapid methodology of E-RT-MCDA was developed for the detection of ML. A previous study published that the LoD of E-RT-MCDA was 10- and 100-fold more sensitive than that of qPCR and PCR methods, respectively [23].

The MCDA method was first established by Wang et al. in 2015 and shows advantages over the conventional PCR technique in that it is rapid, robust, specific and sensitive [17]. In this study, the MCDA products were confirmed through three determinations, including colorimetric indicators, gel electrophoresis and PCR sequencing. Finally, the sequencing data showed that the correct amplification of MCDA was 98% concordant with the expected sequence [17]. To be more valuable and widely applicable, endonuclease restriction-mediated real-time multiple cross displacement amplification (E-RT-MCDA) was developed and validated [23]. The methodology of E-RT-MCDA with 10 sequence-specific primers, which recognised 10 regions of approximately 200 bp for the target gene, provided high specificity [17, 23]. Moreover, restriction endonucleases (e.g., BstUI and Nb.*BsrDI*), with their specific cleavage of the double-stranded recognition sites, were revealed as a novel strategy for real-time detection of nucleic acid sequences [23, 24]. To achieve rapid detection and real-time testing of leprosy, the restriction endonuclease (Nb.*BsrDI*) cleavage and multiple cross displacement amplification strategies were integrated into a one-tube reaction, which requires no additional probe design or testing, and allows real-time detection [19, 23].

In the current study, the target sequence was located on the RLEP gene, which belongs to a family of dispersed repeats (including RLEP1, RLEP2, RLEP3*,* and RLEP4) in ML with an unknown function [14, 25, 26]. The RLEP element consists of a 545 bp core flanked in some cases by additional segments ranging from 44 to 100 bp [26, 27]. The ML repetitive element RLEP is considered to be highly sensitive, 100% specific, and a suitable target for diagnostic applications [5, 28–30]. RLEP sequences have also been widely used for ML detection [9, 12–14, 31]. RLEP2 covers the central domain of the RLEP element [27] and was selected as the target gene for ML detection in this study. To be more precise, we designed three sets of MCDA primers for the RLEP sequence and used a real-time turbidimeter (LA-500) to find the most efficient primer. In the selected MCDA primers, RLEP-D1* was joined to a short sequence (5'-TGCAATG-3') at the 5’ end, which can be recognised by Nb.*BsrDI* enzyme (5'-GCAATGNN-3', N = A, G, C and T) [23]. The thymine (T) at the 5’ end can act as a protective base [24]. FAM was then labeled at the 5’ end of the RLEP-D1* primer, and the dark quencher BHQ1 was also integrated into the primer sequence. Nb.*BsrDI* was employed to cleave the labeled sequence, and release fluorescein (FAM) from the dark quencher (BHQ1) (Fig. 6). Furthermore, to investigate the efficiency of the E-RT-MCDA reaction, three methods (agarose gel electrophoresis, real-time turbidimetry and real-time PCR) were used to report the amplification products (Fig. 1). The results indicated that the MCDA primers for the amplification of RLEP were credible.

For the temperature optimization of the reaction system, we set the temperature range from 65 °C to 69 °C for the RLEP sequence, which has high efficiency in the early reaction within 20 min. A temperature of 67 °C showed the highest efficiency for RLEP amplification (Fig. 2). Additionally, after 36 min of amplification, both the real-time turbidity and the accumulation turbidity had reached a plateau. Therefore, the detection was completed within 36 min with a rapid reaction, and we chose 67 °C for 36 min as the optimal reaction conditions in the subsequent study. In addition, the serial dilutions of ML genomic DNA were used to analyze the LoD. Surprisingly, the sensitivity of the RLEP gene was 48.6 fg in per reaction (Fig. 3A). Importantly, we found that the E-RT-MCDA assay was more sensitive than the conventional PCR method, and reaching up to 100-fold. Furthermore, the CT was plotted against the DNA concentration of the reactions, providing a reference for detection of the clinical samples (Fig. 3B). The positive CT values of the samples were within the linear range (Table S1). We then employed 26 pathogenic bacterial strains (including 16 Mycobacterium strains and 10 non-Mycobacterium strains) to verify the specificity, and ML genomic DNA was used as a positive control (PC) (Table 1). The results showed that the specificity of the E-RT-MCDA system was as high as 100% (Fig. 4). Thus, the novel developed E-RT-MCDA assay was rapid, sensitive, available and reliable for the detection of ML.

Generally, the WHO classification of leprosy uses paucibacillary (PB) and multibacillary (MB), where the leprosy patients with fewer than five skin lesions are defined as PB and those with more than five are defined as MB [5, 32]. Although treatment for leprosy is available, access is limited, control is suboptimal unless started early in the course of the disease, and there may be permanent disabling effects [33]. There is evidence to suggest that early diagnosis could prevent transmission and contribute to epidemiological control [34, 35]. In the current study, a total of 45 clinical leprosy samples were collected and classified into 7 PB cases and 38 MB cases (Table S1). To evaluate the practical applicability, E-RT-MCDA and conventional PCR methods were employed to detect leprosy samples individually. The data showed that for the detection of PB samples, the detection rate of conventional PCR was only 57.14% (4/7), while the rate in the E-RT-MCDA system was as high as 71.43% (5/7). The E-RT-MCDA system also showed a higher rate of detection for skin tissue fluid samples at 86.67% (13/15) compared to 60.00% (9/15) for conventional PCR (Table 2). In this study, the application samples were limited, and PB cases were rare. Nevertheless, the sensitivity test showed a lower LoD (48.6 fg per vessel), and it was more suitable for the low bacterial content, such as the PB cases and early leprosy cases. In addition, a few samples were not detected, possibly due to the multidrug therapy (MDT) of the leprosy patients. Compared to E-RT-MCDA, PCR-based detection requires more instrumentation (e.g., the gel electrophoresis apparatus and UV imaging equipment) [5, 9, 12]. The novel E-RT-MCDA system performed satisfactorily with a higher sensitivity and had a wider range of application than the conventional PCR system. In short, the E-RT-MCDA system was an isothermal amplification technique that not only overcame the shortcomings of the PCR method, but also had the advantages of high sensitivity.

## Conclusion

Here, we report a reliable E-RT-MCDA system for the detection of ML by targeting the RLEP gene. This novel tool was developed by combining the restriction endonuclease cleavage and real-time fluorescence monitoring. MCDA provided a rapid isothermal amplification, allowing detection to be completed within 36 min. All the data showed that the developed E-RT-MCDA method had excellent ML detection capacity with high sensitivity and specificity. Overall, the E-RT-MCDA system provides a more reliable, sensitive and rapid technique for the detection of ML and could be a potentially valuable tool in the clinical early diagnosis of leprosy.

## Materials and methods

### Reagents and Apparatus

DNA isothermal amplification kits and Nb.*BsrDI* was purchased from Bei-Jing Hai Tai Zheng Yuan Co., Ltd. (Beijing, China). The Bacterial Genomic DNA Extraction Kit was provided by Tianlong Technology Co., Ltd. (Xi’an, China).

### Design and synthesis of MCDA primers

**Fig. 5:**
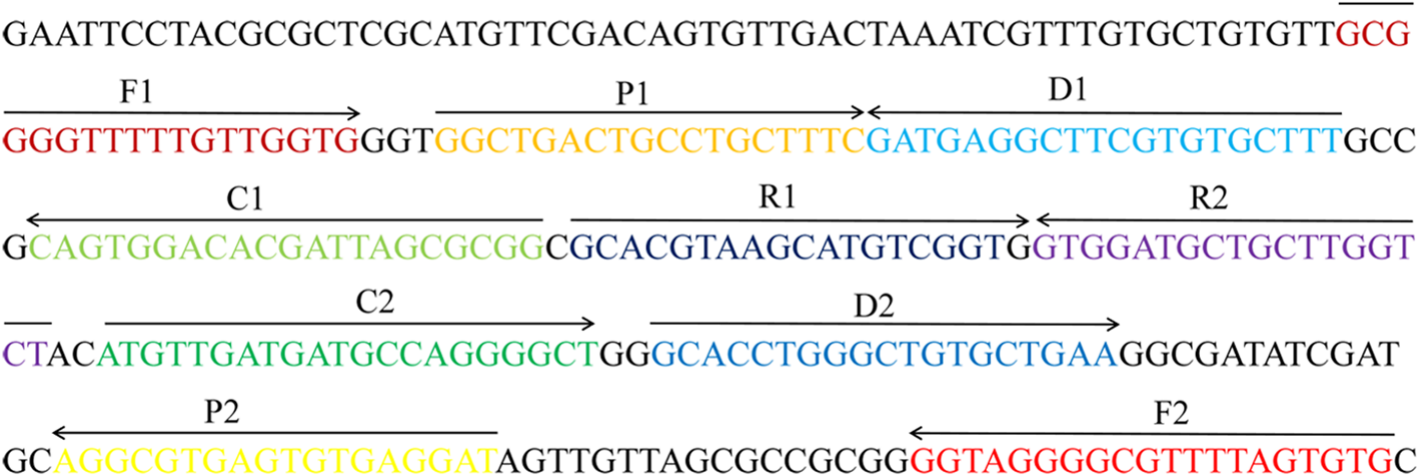
Primer design for the ML E-RT-MCDA method. The nucleotide sequence and location of the RLEP gene used to design the ML E-RT-MCDA primers. Right arrows and left arrows indicate the sense and complementary sequences that are designed. MCDA, multiple cross displacement amplification

According to the reaction mechanism of MCDA, a set of primers was designed against the target RLEP gene (GenBank accession no. X17151.1 ML repetitive element, RLEP2). The primers included displacement primers F1 and F2; amplification primers C1, C2, R1, R2, D1 and D2; and cross primers CP1 and CP2, which were designed using software (PrimerExplorer V5, Eiken Chemical). Moreover, 6-carboxy-fluorescein (FAM) was labeled at the 5’ end of the RLEP-D1 primer, and BHQ1 was used as a dark quencher. Additionally, the cross primers CP1 and CP2 were joined through P1 and C1 and P2 and C2, respectively. Details of the primers, including sequences, locations and modifications, are shown in Fig. 5 and Table 3. The target sequence primers were synthesized and purified to HPLC grade by Biotech Co., Ltd. (Tianyi-Huiyuan, Beijing, China).

**Table 3:** The details of primers for the RLEP gene

Gene^a^	Primers name	Sequences and modifications^b^	Length^c^
**RLEP**	RLEP-F1	5’-GCGGGGTTTTTGTTGGTG-3’	18 nt
	RLEP-F2	5’-CACACTAAAACGCCCCTACC-3’	20 nt
	RLEP-CP1	5’-CCGCGCTAATCGTGTCCACTG-GGCTGACTGCCTGCTTTC-3’	39 mer
	RLEP-CP2	5’-ATGTTGATGATGCCAGGGGCT-ATCCTCACACTCACGCCT-3’	39 mer
	RLEP-C1	5’-CCGCGCTAATCGTGTCCACTG-3’	21 nt
	RLEP-C2	5’-ATGTTGATGATGCCAGGGGCT-3’	21 nt
	RLEP-D1*	5’-FAM-TGCAATG-AAAGCACACGAAGCCT(BHQ1)CATC-3’	27 nt
	RLEP-D2	5’-GCACCTGGGCTGTGCTGAA-3’	19 nt
	RLEP-R1	5’-GCACGTAAGCATGTCGGT-3’	18 nt
	RLEP-R2	5’-AGACCAAGCAGCATCCAC-3’	18 nt

^a^RLEP, the repetitive element gene of ML; ^b^FAM, 6-carboxy-fluorescein; BHQ1, black hole quencher 1; ^c^mer, monomeric unit; nt, nucleotide

### Preparation of samples

The experiments involved 27 bacterial pathogens and 45 clinical samples from leprosy patients (including 15 skin tissue fluid samples and 30 skin tissue samples). The ML DNA was provided by the Institute of Dermatology, Chinese Academy of Medical Sciences (CAMS), and was obtained from the foot pads of infected mice. The other DNA was obtained from the Guizhou Provincial Center for Disease Control and Prevention, China (GZCDC) (Table 1). In addition, the clinical samples were collected by the GZCDC and stored in 70% ethanol solution at -80 °C.

### DNA extraction of specimens

The strains were inactivated in a metal bath at 80 °C for 30 min with 0.5 mL of 1 × TE. The skin tissue samples were cut into pieces and ground to homogeneity. All specimens (including skin tissue fluid samples) were centrifuged at 12,000 rpm for 5 min (centrifuge radius was 7.5 cm), and the supernatant was removed. Genomic DNA was then extracted using the Bacterial Genomic DNA Extraction Kit (Tianlong Technology Co., Ltd. Xi'an, China). In short, the processed specimens were treated with 20 μl protease K and 180 μl lysozyme for 30 min at 50 °C. Subsequently, 200 µl of bacterial digestion solution was added to the mixtures, and genomic DNA was extracted using an automated nucleic acid extractor. The genomic DNA was stored at -20 °C.

### Standard E-RT-MCDA reaction

The E-RT-MCDA reaction mixtures were contained a final volume of 25 μL, which included 12.5 μl 2 × reaction buffer, 1 μl Bst 2.0 DNA polymerase (10 U), 1 μl Nb.*BsrDI* (10 U), 2.2 μl mixed primers, genomic templates (1 μl for pure ML genomic DNA/2.5 µl for clinical samples) and distilled water (DW). The mixed primers consisted of 0.4 μM each of F1, F2, C1 and C2; 1.2 μM each of R1, R2, D1 and D2; and 2.4 μM each of CP1 and CP2. The mixtures were then reacted at a preset temperature of 65 °C, and three monitoring tools, including a real-time turbidimeter (LA-500), agarose gel electrophoresis and real-time PCR (ABI 7500 Fast), were employed to determine and verify the E-RT-MCDA amplification products. *M. tuberculosis* (H37Rv, ATCC 27294) genomic DNA and double distilled water (DW) were used as negative controls and blank controls, respectively.

### Optimizing the reaction temperature of the E-RT-MCDA assay

The optimal reaction temperature for E-RT-MCDA was determined using ML genomic DNA at a concentration of 1.52 × 10^5^ fg per reaction. The constant reaction temperature ranged from 63 ℃ to 70 ℃ with 1 ℃ intervals. After monitoring the amplicons with a real-time turbidimeter (LA-500) according to the standard MCDA assay, a turbidity > 0.1 (the threshold value was 0.1) was considered as positive amplification [17].

### Verification of sensitivity and specificity of the E-RT-MCDA assays

Fivefold serial dilutions (1.9 × 10^7^ fg–1.95 fg per microliter) of ML genomic DNA were employed to analyze the sensitivity of the E-RT-MCDA assay. The serial dilutions were then used to test the limit of detection (LoD) and replicated 6 times. Additionally, 27 pathogen DNA samples were used to verify the specificity of E-RT-MCDA with the optimized reaction conditions. All products were monitored by the real-time fluorescence detector, and the DW was the blank control.

### Applicability of the E-RT-MCDA assay to clinical samples

The feasibility of the E-RT-MCDA assay was determined with 2.5 µl of DNA templates from different clinical samples. These samples were simultaneously detected by conventional PCR targeting the RLEP gene (approximately 400 bp) [9], which was amplified in a 50 µl mixture containing 25 µl 2 × Taq Master Mix (CoWin Biosciences Co., Ltd. Beijing, China), 0.5 μM each of RLEP-F (5’-CGGCCGGATCCTCGATGCAC-3’) and RLEP-R (5’-GCACGTAAGCTTGTCGGTGG-3’), 5 µl DNA templates of the samples, and DW. The amplification conditions were 94 °C (5 min), 35 cycles of 94 °C (30 s), 58 °C (30 s) and 72 °C (30 s), and a final extension of 10 min. The reactions were then subjected to an automated thermal cycler (Thermo Fisher Scientific Co., Ltd. USA). The PCR products were analyzed under UV light (Bio-Rad, USA) by using a 1.5% agarose gel (containing GelGreen dye). *M. tuberculosis* genomic DNA was used as a negative control (NC), and DW was used as a blank control (BC).

### Schematic mechanism of the E-RT-MCDA assay

**Fig. 6:**
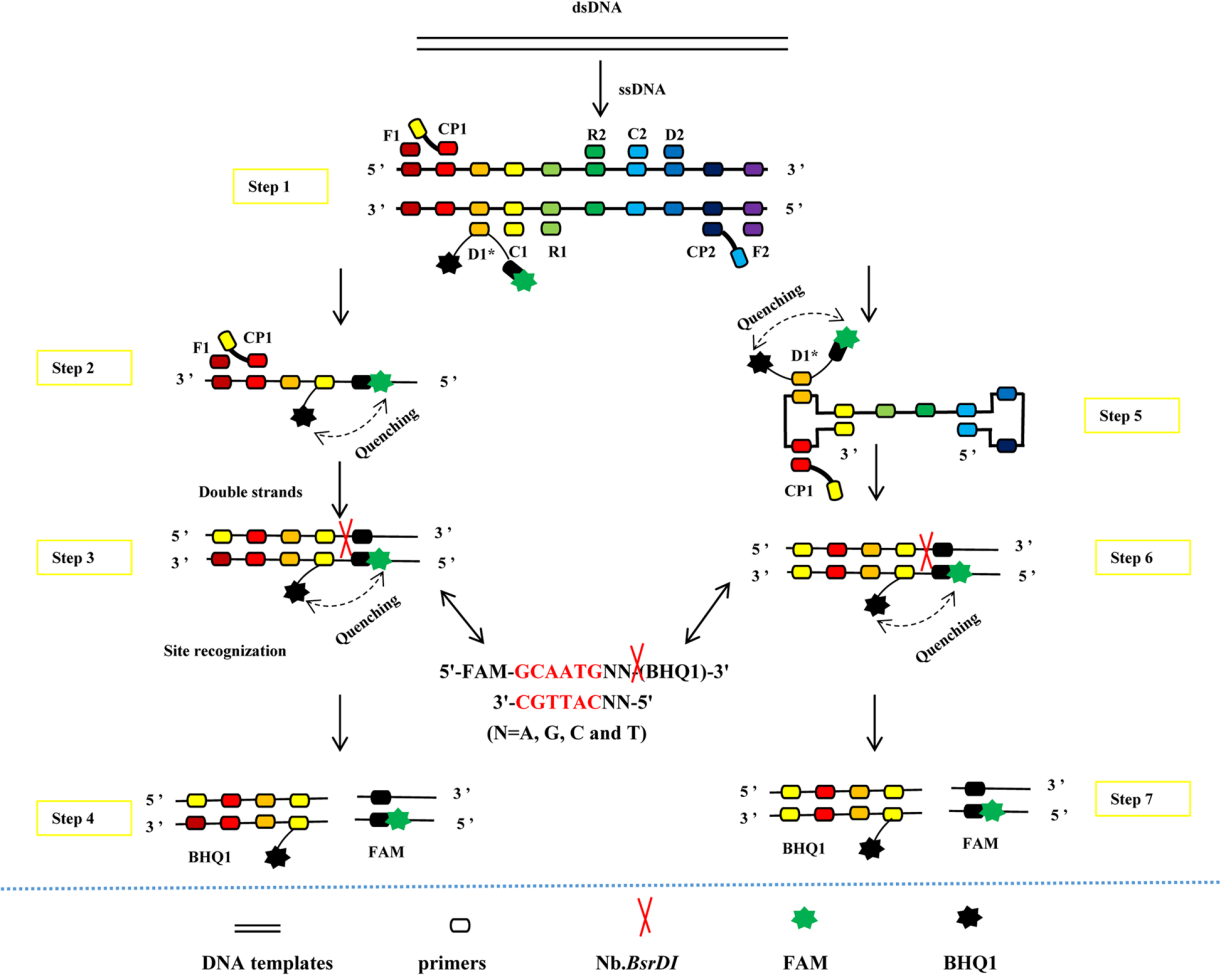
Outline of the E-RT-MCDA assay. There are seven steps in this schematic: Step 1, at a constant temperature, the double-stranded DNA becomes scattered single strands, and the different primer binding sites are highlighted. Steps 2 and 5, the ssDNA strands (including sense strands and antisense strands) as templates are amplified in the MCDA reaction system. Steps 3 and 6, at the start of amplification, the short sequence (5'-TGCAATGNN-3') is recognized and cleaved by the Nb.BsrDI enzyme. Steps 4 and 7, the cleavage causes the fluorescein groups (FAM) and the dark quencher (BHQ1) to separate from the two sides of the short sequences, and the fluorescent signals are collected by a real-time fluorescence detector

In the E-RT-MCDA system, a key primer (RLEP-D1*) targeting the RLEP gene with restriction enzyme recognition sites for Nb.*BsrDI* was designed and labeled at the 5’ end with fluorescein (FAM), and the corresponding dark quencher (BHQ1) was inset in the middle of the primer. In the constant temperature reaction mixture, the double-stranded DNA became scattered single strands, and the target primers were attached to the single strands, and amplification began. When the labeled double strands were synthesized, the Nb.*BsrDI* recognized the specific sites and cleaved the sequences. Instantaneously, the fluorescein groups (FAM) and dark quenchers (BHQ1) were separated and the fluorescent signals were collected by a real-time fluorescence detector. The schematic reaction mechanism of the E-RT-MCDA assay is shown in Fig. 6.

### Supplementary Information

**Additional file 1.**

## Data Availability

The datasets used and/or analyzed during the current study are available from the corresponding author upon reasonable request. The raw sequence data reported in this paper were obtained GenBank, accession No. X17151.1 ML repetitive element, RLEP2 (https://www.ncbi.nlm.nih.gov/nuccore/X17151.1/).

## Abbreviations

ML: *Mycobacterium leprae*
MCDA: Multiple cross displacement amplification
AFB: Acid fast bacilli
WHO: World Health Organization
PB: Paucibacillary
MB: Multibacillary
ATCC: American Type Culture Collection
LoD: Limit of detection
GZCDC: Guizhou Provincial Center for Disease Control and Prevention
mer: Monomeric unit
nt: Nucleotide
FAM: 6-Carboxyfluorescein
BHQ1: Black hole quencher 1
P: Positive
N: Negative
DW: Double-distilled water
BC: Blank control
PC: Positive control
NC: Negative control
