# Supplementary material for: Highly sensitive and rapid determination of Mycobacterium leprae based on real-time multiple cross displacement amplification
Source: BMC Microbiol. 2023 Sep 28;23:272. doi: 10.1186/s12866-023-03004-7 (PMC10537127; doi:10.1186/s12866-023-03004-7)
Supplement: Supplementary file 1 — Additional file 1. [file 12866_2023_3004_MOESM1_ESM.pdf]

Supplementary Materials

Highly sensitive and rapid determination of *Mycobacterium leprae* based on real-time multiple cross displacement amplification

Junfei Huang[1], Yi Tong[1], Yijiang Chen[1], Xinggui Yang[1], Xiaoyu Wei[1], Xu Chen[2], Jinlan Li[3], Shijun Li[1, 4*]

[1] Laboratory of Infectious Disease of Experimental Center, Guizhou Provincial Center for Disease Control and Prevention, Guiyang, 550004, Guizhou, P.R. China

[2] The Second Affiliated Hospital, Guizhou University of Traditional Chinese Medicine, Guiyang, Guizhou, 550003, P.R. China

[3] Tuberculosis Control Institute, Guizhou Provincial Center for Disease Control and Prevention, Guiyang, 550004, Guizhou, P.R. China

[4] Public Health School, Guizhou Medical University, Guiyang, 550025, Guizhou, P.R. China.

*** Correspondence:**

Shijun Li

E-mail: zjumedjun@163.com

Table S1. Leprosy samples detail and detection

NO.	City	Types	Classification	PCR	E-RT-MCDA(CT)
S1	Anshun	Skin tissue	MB	+	17.5
S2	Zunyi	Skin tissue	MB	+	15.2
S3	Bijie	Skin tissuefluid	MB	-	-
S4	Bijie	Skin tissuefluid	MB	+	13.9
S5	Bijie	Skin tissuefluid	PB	-	-
S6	Guiyang	Skin tissue	MB	+	11.2
S7	Guiyang	Skin tissue	MB	+	14.3
S8	Anshun	Skin tissue	MB	+	10.5
S9	Anshun	Skin tissuefluid	MB	+	20.1
S10	Guiyang	Skin tissue	MB	+	10.5
S11	Guiyang	Skin tissue	MB	+	12.1
S12	Anshun	Skin tissuefluid	MB	-	13.2
S13	Qiandongnan	Skin tissue	MB	+	11.5
S14	Qiandongnan	Skin tissue	PB	+	16.3
S15	Qiandongnan	Skin tissue	MB	+	13.5
S16	Tongren	Skin tissue	PB	-	-
S17	Bijie	Skin tissuefluid	MB	+	17.5
S18	Tongren	Skin tissuefluid	MB	+	21.5
S19	Bijie	Skin tissuefluid	MB	+	20.3
S20	Bijie	Skin tissuefluid	MB	+	18.6
S21	Tongren	Skin tissuefluid	MB	+	19.4
S22	Bijie	Skin tissuefluid	MB	-	21.7
S23	Bijie	Skin tissuefluid	PB	-	20.2
S24	Bijie	Skin tissuefluid	MB	-	22.3
S25	Bijie	Skin tissuefluid	MB	+	19.5
S26	Bijie	Skin tissuefluid	PB	+	18.7
S27	Qiannan	Skin tissue	PB	+	16.5
S28	Qiannan	Skin tissue	PB	+	15.2
S29	Qiannan	Skin tissue	MB	+	14.5
S30	Qiannan	Skin tissue	MB	+	17.5
S31	Qiannan	Skin tissue	MB	+	16.3
S32	Qiannan	Skin tissue	MB	+	15.0
S33	Qiannan	Skin tissue	MB	+	14.1
S34	Qiannan	Skin tissue	MB	+	19.5
S35	Qiannan	Skin tissue	MB	+	16.2
S36	Qiannan	Skin tissue	MB	+	15.1
S37	Qiannan	Skin tissue	MB	+	15.6
S38	Qiannan	Skin tissue	MB	-	17.5
S39	Qiannan	Skin tissue	MB	+	11.1
S40	Liupanshui	Skin tissue	MB	+	12.0
S41	Qiandongnan	Skin tissue	MB	+	11.5
S42	Qiandongnan	Skin tissue	MB	+	15.0
S43	Qiannan	Skin tissue	MB	+	14.0
S44	Qiandongnan	Skin tissue	MB	+	13.5
S45	Qiandongnan	Skin tissue	MB	-	24.0

Abbreviations: PCR, polymerase chain reaction; E-RT-MCDA, multiple cross displacement amplification combined with endonuclease restriction-mediated real-time PCR; MB, multibacillary; PB, paucibacillary.

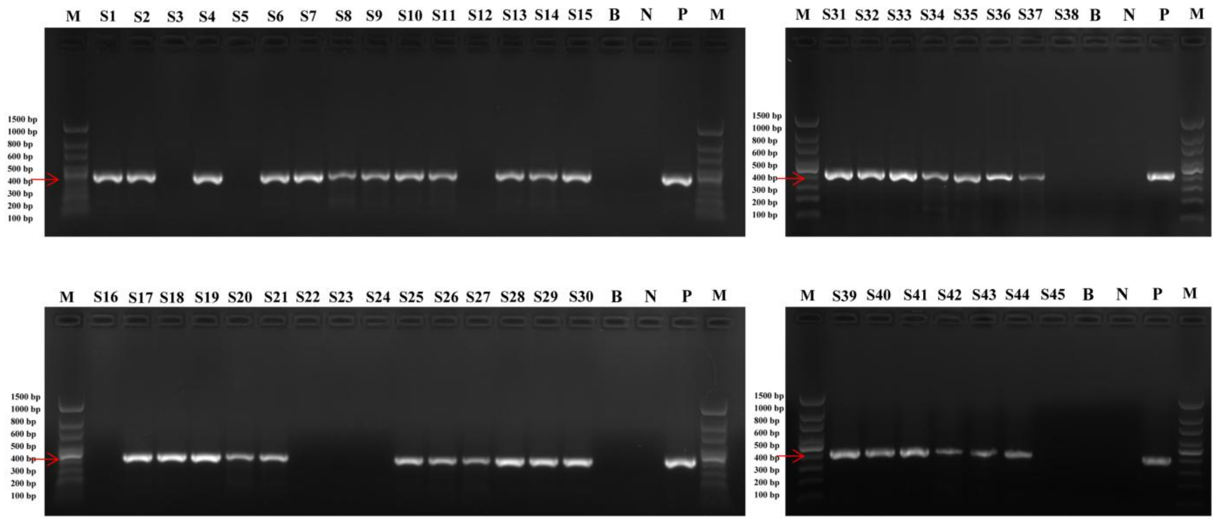

Fig. S1 The conventional PCR targeting the RLEP gene for detection of leprosy clinical samples. **M** is the marker ladder from 100 bp to 1500 bp. **S1-S45** are the leprosy clinical samples, the details have been shown in **Table S1**. **B** is the blank control (BC) with double distilled water (DW). **N** is the negative control (NC) containing with *M. tuberculosi*s genomic DNA.
